# Tissue Degeneration following Loss of *Schistosoma mansoni cbp1* Is Associated with Increased Stem Cell Proliferation and Parasite Death *In Vivo*

**DOI:** 10.1371/journal.ppat.1005963

**Published:** 2016-11-03

**Authors:** Julie N. R. Collins, James J. Collins

**Affiliations:** Department of Pharmacology, UT Southwestern Medical Center, Dallas, Texas; George Washington University School of Medicine and Health Sciences, UNITED STATES

## Abstract

Schistosomiasis is second only to malaria in terms of the global impact among diseases caused by parasites. A striking feature of schistosomes are their ability to thrive in their hosts for decades. We have previously demonstrated that stem cells, called neoblasts, promote homeostatic tissue maintenance in adult schistosomes and suggested these cells likely contribute to parasite longevity. Whether these schistosome neoblasts have functions independent of homeostatic tissue maintenance, for example in processes such as tissue regeneration following injury, remains unexplored. Here we characterize the schistosome CBP/p300 homolog, *Sm-cbp1*. We found that depleting *cbp1* transcript levels with RNA interference (RNAi) resulted in increased neoblast proliferation and cell death, eventually leading to organ degeneration. Based on these observations we speculated this increased rate of neoblast proliferation may be a response to mitigate tissue damage due to increased cell death. Therefore, we tested if mechanical injury was sufficient to stimulate neoblast proliferation. We found that mechanical injury induced both cell death and neoblast proliferation at wound sites, suggesting that schistosome neoblasts are capable of mounting proliferative responses to injury. Furthermore, we observed that the health of *cbp1(RNAi)* parasites progressively declined during the course of our *in vitro* experiments. To determine the fate of *cbp1(RNAi)* parasites in the context of a mammalian host, we coupled RNAi with an established technique to transplant schistosomes into the mesenteric veins of uninfected mice. We found transplanted *cbp1(RNAi)* parasites were cleared from vasculature of recipient mice and were incapable of inducing measurable pathology in their recipient hosts. Together our data suggest that injury is sufficient to induce neoblast proliferation and that *cbp1* is essential for parasite survival *in vivo*. These studies present a new methodology to study schistosome gene function *in vivo* and highlight a potential role for schistosome neoblasts in promoting tissue repair following injury.

## Author Summary

Schistosomes are parasitic flatworms that infect more than 200 million people in the developing world. Once these parasites infect a human, they are capable of living in the bloodstream for decades. Previously, our group has shown that these parasites have stem cells that are capable of renewing worn out cells in these parasites. Although we know these stem cells can continuously restore aging tissues, whether these stem cells can respond to injuries in the parasite is not clear. Here, we show that reducing levels of a gene called *cbp1* results in cell death and degeneration of certain schistosome tissues and causes dramatic increases in the level of stem cell proliferation. This result suggested that the parasites might perceive elevations in cell death as a form of injury, which then triggers stem cell proliferation to enhance the rate of tissue repair. To explore this idea further, we physically injured parasites and observed that physical injury was indeed capable of inducing stem cell proliferation. We also found that loss of *cbp1* resulted in the progressive decline in the health of parasites cultured in the laboratory, likely as a result of increased cell death and tissue degeneration. To determine if *cbp1* was also important for parasites living inside a mammalian host, we coupled our gene disruption approaches with a classic technique to transplant schistosomes into the veins of uninfected mice. Using this novel methodology, we found that reducing *cbp1* levels resulted in parasite death inside mice. Taken together, these observations demonstrate that *cbp1* is important for parasite survival inside a mammalian host and show that schistosome stem cells are capable of responding to injury. These data have important implications for the development of new therapies and for understanding the roles stem cells play in the biology of schistosome infection.

## Introduction

Schistosomes infect over 200 million people and are a major cause of morbidity in the developing world. The primary driver of this morbidity is the prodigious egg production of these parasites, which can lay several hundred eggs every day while living in the vasculature of their hosts [1]. A large fraction of these eggs are swept into the circulation and become lodged in host organs (such as the liver and bladder), leading to inflammatory responses that can compromise organ function [2]. The pathological consequences of schistosome egg production are compounded by the fact that schistosomes can survive and produce eggs for decades inside their human hosts [1, 3]. Understanding the developmental forces that promote parasite longevity is essential for understanding the chronic nature of this disease.

Schistosomes possess a population of somatic stem cells similar to the neoblasts found in free-living flatworms (e.g., freshwater planarians) [3, 4]. In schistosomes, these neoblast-like cells appear to represent the only proliferative somatic cell type [4] and support the homeostatic renewal of tissues such as the intestine [4] and tegument [5]. Together, these data suggest that schistosome neoblasts are likely critical for long-term parasite survival in their hosts. What is not clear is whether neoblasts serve other important functions in these parasites. In free-living planarians, neoblasts are essential for both homeostatic tissue maintenance and for tissue regeneration [6, 7]. Following amputation, there is a burst in planarian neoblast proliferation, which fuels the regeneration of damaged and missing tissues [8, 9]. Unlike planarians, schistosomes live exclusively in the vasculature of mammalian hosts and are unlikely to face the same types of mechanical insults (e.g., amputation) that planarians do [3]. Therefore, whether schistosome neoblasts are capable of interpreting injury signals and modulating their behavior to repair damage is not clear. However, since schistosomes are likely subjected to a myriad of immunological and chemical insults inside their mammalian host, it is possible that neoblasts could possess the capacity to respond to various types of injury. Thus, understanding how parasites respond to injury, and the role of neoblasts in tissue repair, would provide important new insights into the mechanisms that support parasite longevity *in vivo*.

During the course of a systematic effort to identify factors with the potential to regulate schistosome neoblast function we characterized *Sm-cbp1* (for brevity, we will refer to this gene as *cbp1*), a gene that encodes a homolog of the mammalian CBP/p300 family of proteins [10]. In mammals, these proteins serve as transcriptional co-activators that possess histone acetyltransferase (HAT) activity [11]. In schistosomes, *cbp1* was previously demonstrated to act as a transcriptional co-activator *in vitro* [10] and suggested to regulate genes important for schistosome egg production via its HAT activity [12]. Here we show that abrogation of *cbp1* function leads to simultaneous increases in cell death and neoblast proliferation. Based on our observation that physical injury similarly induces parasite cell death and neoblast proliferation, we suggest that increases in neoblast proliferation following *cbp1(RNAi)* is a strategy by the parasite to cope with cell death-mediated tissue damage. In addition, we report a novel application of existing techniques to examine adult schistosome gene function *in vivo* and show that *cbp1* is essential for parasite survival in mice. These data suggest an important function for *cbp1* in parasite survival and highlight a potential role for neoblasts in regenerative processes in schistosomes.

## Results

### Depletion of *cbp1* levels by RNA interference (RNAi) results in increased neoblast proliferation

**Fig 1:**
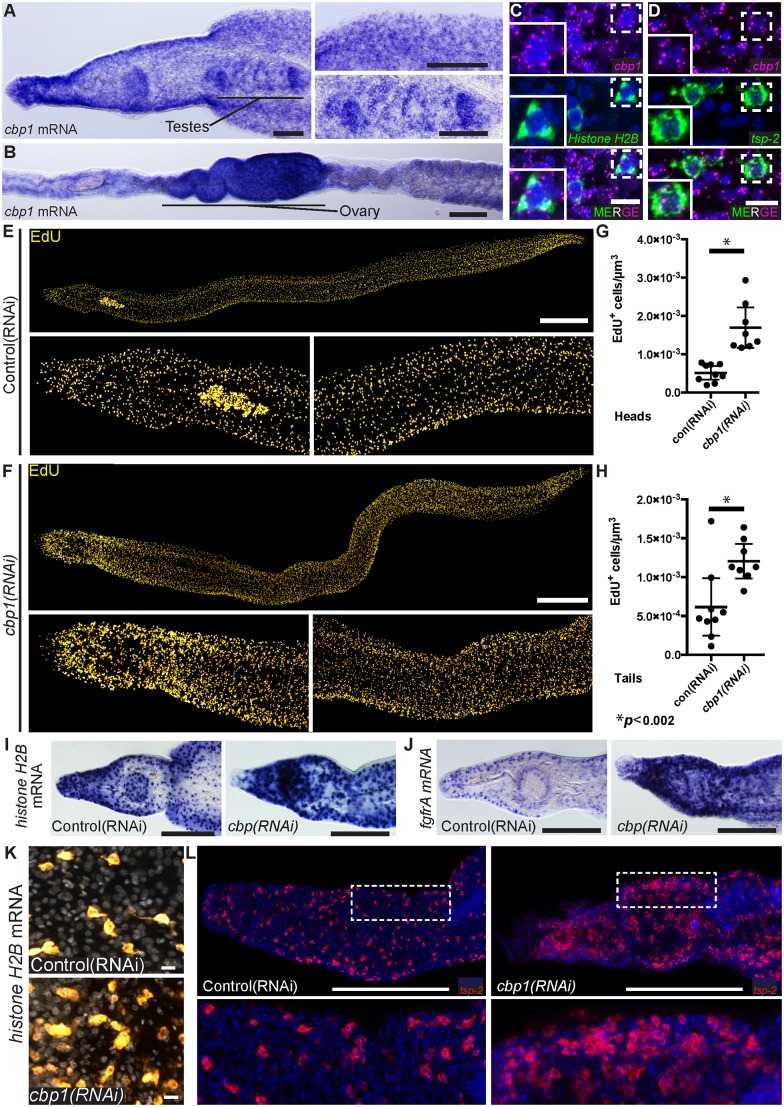
*cbp1(RNAi)* results in an increase in neoblast proliferation. (A, B) Whole mount in situ hybridization showing *cbp1* mRNA expression in (A) male and (B) female parasites. *cbp1* is expressed broadly in the worm including in the testes and ovaries. (C, D) FISH showing expression of *cbp1* with (C) *Histone H2B* and (D) *tsp-2*. Nuclei are in blue. (E, F) EdU labeling showing cell proliferation in (E) control and (F) *cbp1(RNAi)* male parasites. Images are tiled from multiple confocal stacks acquired from parasites at D14 of RNAi. Parasites were fixed after an overnight EdU pulse. (G,H) Quantification of EdU^+^ neoblasts per μm^3^ from (G) heads and (H) tails. Each dot represents counts from a confocal stack taken from a single male parasite and error bars represent 95% confidence intervals. Differences are statistically significant (p < 0.002, t-test). Quantification was performed on male parasites at D14 of RNAi. (I,J) WISH for neoblast markers (I) *Histone H2B* and (J) *fgfrA* in control and *cbp1(RNAi)* animals. Images are from D14 of RNAi and are representative of n > 10 male parasites. (K) FISH for *Histone H2B* in control and *cbp1(RNAi)* animals. Nuclei are in gray. Images are from D11 of RNAi. (L) FISH for *tsp-2* in control and *cbp1(RNAi)* animals. D13 of RNAi. Anterior faces left in A-B, E-F, I, J, and L. Scale Bars: A,B 100 μm; C,D, and K 10 μm; E,F 500 μm; I,J, and L 250 μm.

Using whole-mount in situ hybridization (WISH) we found that *cbp1* was expressed in adult parasites in a variety of cells throughout the worm’s parenchyma and in cells within the male testes and female ovaries (Fig 1A and 1B). To characterize how broadly *cbp1* was expressed in the parenchyma, we performed fluorescence in situ hybridization (FISH) with two markers of somatic cells residing in the parenchyma: *Histone H2B* to mark neoblasts [4] and *tsp-2* to label tegument-associated cells [5]. In addition to being expressed in both *Histone H2B* ^+^ and *tsp-2* ^+^ cells, we weakly detected *cbp1* transcripts in most cells within the schistosome parenchyma (Fig 1C and 1D). While we cannot conclude that *cbp1* is expressed in every cell in the worm, our data suggest this gene is expressed in a large number of schistosome cell types.

To explore a role for *cbp1* in regulating schistosome stem cells, we performed RNAi experiments. In comparison to controls, depletion of *cbp1* mRNA levels (S1A and S1B Fig) led to a dramatic (Fig 1E and 1F) and statistically significant (Fig 1G and 1H) increase in the number of neoblasts that incorporated the thymidine analog EdU. Similar increases in cell proliferation were observed with dsRNAs targeting two distinct regions of the *cbp1* gene, indicating these effects are specific to the reduction of *cbp1* levels (S1A and S1C Fig) and not due to off-target effects. To explore this observation further, we also performed WISH with the neoblast markers *Histone H2B* and *fgfrA* [4] (Fig 1I and 1J) and FISH (Fig 1K) with *Histone H2B*. Similar to our observations with EdU incorporation, we noted an increase in the number of cells expressing neoblast markers (Fig 1I–1K). Together, these data suggest that loss of *cbp1* increases the number of proliferative neoblasts.

Two simple stem cell behaviors can explain our observations following *cbp1(RNAi)*. First, loss of *cbp1* could block the ability of neoblasts to differentiate, effectively locking the cells in a proliferative state. This type of behavior is observed following perturbations that block planarian neoblast differentiation [13]. Alternatively, the cells could maintain the capacity for differentiation but the size of the stem cell pool is expanded via an increased rate of cell proliferation. To distinguish between these possibilities, we performed WISH for the neoblast differentiation progeny marker *tsp-2*. Previously we demonstrated that *tsp-2* is expressed in a tegument-associated cell population that is the primary differentiation progeny of schistosome neoblasts [5]. Since *tsp-2* ^+^ cells are short lived and rapidly renewed by neoblasts [5], they are a sensitive measure of the capacity for neoblasts to differentiate. Consistent with neoblasts in *cbp1(RNAi)* parasites maintaining the ability to differentiate, we observed substantial increases in the number of *tsp-2* ^+^ cells in *cbp1(RNAi)* parasites (Fig 1L). Together, these data suggest that loss of *cbp1* expands the size of the neoblast pool and this results in an increased rate of production of at least one differentiated cell type.

### Local increases in neoblast proliferation accompany degeneration of the esophageal gland

**Fig 2:**
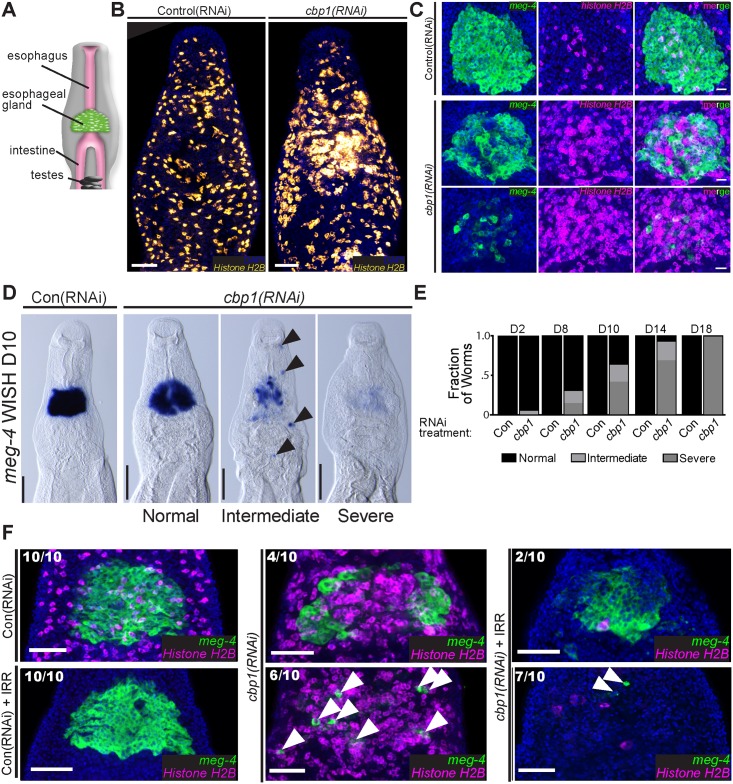
*cbp1(RNAi)* results in degeneration of the esophageal glands. (A) Cartoon depicting the position of the schistosome esophageal gland relative to other tissues. (B) FISH for *Histone H2B* showing a mass of proliferative neoblasts in proximity to the esophageal glands at D11 of RNAi treatment. (C) Double FISH for the esophageal gland marker *meg-4* and the neoblast marker *Histone H2B* following 11 days of control or *cbp1* RNAi treatments. The number of esophageal gland cells is reduced and the number of proliferative neoblasts in increased. In extreme cases only a few *meg-4* ^+^ cells remain in *cbp1(RNAi)* animals. Top and bottom panels for *cbp1(RNAi)* depict animals with differing phenotypic severities. Nuclei are in blue. (D) WISH for *meg-4* at D10 of RNAi treatment. At this time point, by WISH three distinct phenotypic severities are observed: normal, intermediate, and severe. Normal animals possess relatively normal gland structure and *meg-4* ^+^ labeling. Intermediate animals have clearly abnormal or degenerated gland structure and often possess ectopic *meg-4* ^+^ cells (arrowhead) outside of the region were the gland resides in control animals. Severe animals possess few, if any, *meg-4* ^+^ cells. (E) Plots depicting the relative fraction of animals that display normal, intermediate, or severe phenotypes with respect to the esophageal glands as observed by WISH for *meg-4* at D2–D18 of RNAi. >12 animals from two separate experiments were observed for each time point. (F) Double FISH for *Histone H2B* and *meg-4* at D11 in irradiated (+IRR) and unirradiated worms. Degeneration of esophageal glands in *cbp1(RNAi)* worms is not abrogated following irradiation. However, notably more isolated *meg-4* ^+^ cells (Arrowheads) remain in unirradiated *cbp1(RNAi)* worms. Numbers indicate fraction of worms displaying phenotypes similar to those pictured. Nuclei are in blue. Anterior faces up in all images. Scale Bars: B, F 50 μm; C 20 μm; D 100 μm.

The schistosome esophageal glands are located anterior to the intestine (Fig 2A) and are thought to secrete factors that aid in the digestion of blood cells [14, 15]. By both EdU labeling (S1C Fig) and FISH for *Histone H2B* (Fig 2B) we noted a focus of proliferative neoblasts in the vicinity of the esophageal glands in *cbp1(RNAi)* animals. We explored this observation more closely by double FISH for *Histone H2B* and the esophageal gland marker *meg-4* [16, 17]. Consistent with our prediction, at D11 of RNAi-treatment, masses of neoblasts are observed surrounding the esophageal gland of *cbp1(RNAi)* parasites (Fig 2C). In some cases we observed “holes” in the esophageal gland that were occupied by *Histone H2B* ^+^ neoblasts (Fig 2C, top *cbp1(RNAi)* panels). In the most severe cases, the esophageal glands were degenerated and only small numbers of *meg-4* ^+^ cells remained (Fig 2C, bottom *cbp1(RNAi)* panels). To explore the degeneration of the esophageal glands in more detail, we performed time course analyses examining the expression of *meg-4* by WISH. We observed a progressive degeneration of the esophageal gland in *cbp1(RNAi)* parasites and by D18 *cbp1(RNAi)* parasites possessed few traces of *meg-4* ^+^ gland cells (Fig 2D and 2E).

We next explored the relationship between neoblast proliferation and the degeneration of the esophageal glands in *cbp1(RNAi)* parasites. In principle, the observed masses of neoblasts (Fig 2B and 2C) could either be a cause of esophageal gland degeneration, an effect of this degeneration, or unrelated to the disappearance of the gland. Given how prominent the masses of proliferative neoblasts are surrounding the gland (Fig 2B and 2C), we believe the latter of these possibilities is unlikely. Therefore, to determine if neoblast proliferation is a cause or an effect of gland degeneration, we treated parasites with γ-irradiation, which rapidly depletes neoblasts [4], and examined *meg-4* expression by FISH. In control(RNAi) parasites, neoblast depletion had no observable effect on the morphology of the esophageal gland at D11 (Fig 2F). In contrast to control(RNAi) parasites, irradiated and unirradiated *cbp1(RNAi)* parasites displayed extensive degeneration of the esophageal glands (Fig 2F), suggesting that neoblast over proliferation is not likely a direct cause of gland loss. Although we observed substantial gland degeneration in both irradiated and unirradiated *cbp(RNAi)* parasites, we noted more scattered *meg-4* ^+^ cells in unirradiated *cbp1(RNAi)* where neoblasts were present (arrowheads, Fig 2F). Based on this observation we speculate that many of these remaining *meg-4* ^+^ cells in unirradiated *cbp1(RNAi)* parasites represent newly born differentiation progeny of the neoblasts.

### *cbp1(RNAi)* results in elevations of cell death

**Fig 3:**
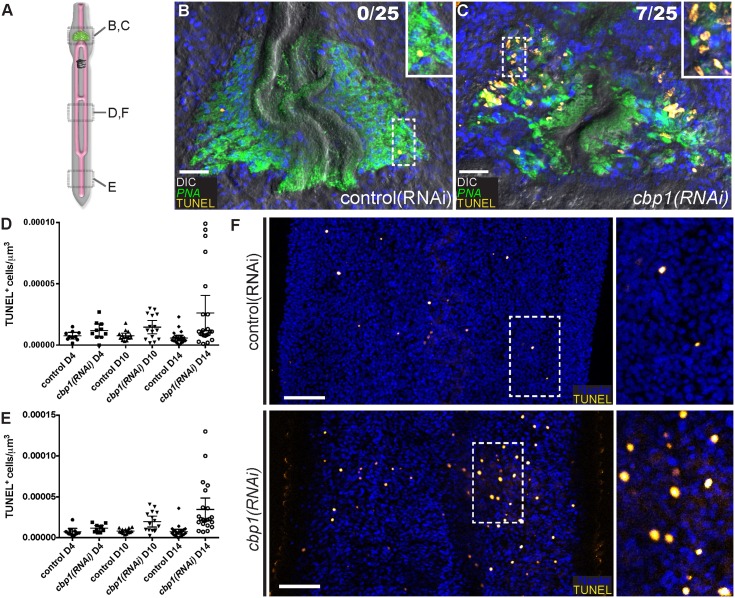
*cbp1(RNAi)* leads to an elevation of cell death in degenerating esophageal glands and throughout the bodies of male parasites. (A) Cartoon showing approximate positions of parasites examined for panels B-F. (B, C) TUNEL in the esophageal glands of (B) control(RNAi) or (C) *cbp1(RNAi)* parasites at D10 of treatment. The lectin PNA (green) is used as a marker of the esophageal gland. At this time point 28% of *cbp1(RNAi)* parasites displayed large number of TUNEL^+^ cells in the region around the esophageal gland. Numbers indicate fraction of animals observed with large numbers of TUNEL^+^ cells within the region of the esophageal gland. Insets are magnified views of the boxed regions. Nuclei are in blue. (D, E) Quantification of TUNEL^+^ cells per μm^3^ from (D) trunks and (E) tails. Each dot represents counts from a confocal stack taken from a single male parasite and error bars represent 95% confidence intervals. Differences are statistically significant at D10 and D14: D10 trunks (p < 0.02, t-test); D10 Tails (p < 0.001, t-test), D14 trunks (p < 0.005, t-test), and D14 tails (p < 0.0002, t-test). At least ten male parasites were examined at each time point. (F) TUNEL staining in control and *cbp1(RNAi)* parasites at D10 of RNAi. *cbp1(RNAi)* results in elevations in cell death relative to controls. Insets are magnified views of the boxed regions. Anterior faces up in all images. Scale Bars: B, C 20 μm; F, 50 μm.

Our data indicated that between D8 and D14 a large fraction of parasites had esophageal glands that were in intermediate stages of degeneration (Fig 2D and 2E). To determine if programmed cell death was playing a role in this degeneration, we developed a whole-mount assay to examine Terminal deoxynucleotidyl transferase dUTP Nick-End Labeling (TUNEL). TUNEL is a methodology to detect double stranded breaks in the DNA of cells undergoing the process of programmed cell death [18], and has been successfully used to detect apoptosis in both free-living flatworms [19] and in sectioned adult female schistosomes [20]. Using this assay we determined that at D10 28% of *cbp1(RNAi)* parasites had large clusters of TUNEL^+^ cells within their esophageal glands (Fig 3A–3C). Visualizing glands with the lectin PNA [21], large pockets of TUNEL^+^ cells were not observed in *cbp1(RNAi)* parasites with largely intact glands nor in parasites with severely degenerated glands. Rather the presence of large numbers of TUNEL^+^ cells was restricted to glands that appeared to be in the early to intermediate stages of degeneration. These data suggest that programmed cell death is a likely driver of esophageal gland cell loss.

In the esophageal glands of *cbp1(RNAi)* parasites we noted elevations in cell proliferation and cell death at roughly similar time points after beginning dsRNA treatment (Figs 2C and 3C). Since we also noted increases in cell proliferation throughout the bodies of *cbp1(RNAi)* parasites (Fig 1F–1H) we explored if cell death was similarly elevated in the trunks and tails of *cbp1(RNAi)* parasites (Fig 3A). Although we did not note measurable changes by D4 of RNAi, at both D10 and D14 we observed statistically significant increases in TUNEL^+^ cells in *cbp1(RNAi)* parasites (Fig 3D–3F); by D14 *cbp1* RNAi treatment on average resulted in 4.6 and 4.8-fold elevations in TUNEL^+^ nuclei in trunks and tails of male parasites, respectively (Fig 3D and 3E). Interestingly, at both D10 and D14 the levels of cell death varied considerably among individual *cbp1(RNAi)* parasites: some *cbp1(RNAi)* parasites possessed levels of TUNEL^+^ nuclei comparable to controls, whereas in other parasites the number of TUNEL^+^ nuclei was dramatically elevated (Fig 3D and 3E). This observation mirrors what we observed in the esophageal glands where large numbers of dying cells were only present in a subset of parasites in which the glands were in the process of degenerating (Fig 3C). Therefore, these elevations in cell death observed in the trunks and tails may similarly reflect the sudden degeneration of one or more tissue types in *cbp1(RNAi)* worms. Unfortunately, given the paucity of cell type-specific markers that are compatible with TUNEL staining, it is presently not possible to determine if this elevated rate of cell death was restricted to a specific cell/tissue type or whether all tissues were undergoing similar levels of cell death. Nevertheless, our data suggest that in addition to being required for preventing cell death, and degeneration of the esophageal gland cells, *cbp1* is important for maintaining normal levels of cell death in other tissues within the parasite.

### Parasite injury induces cell death and subsequent elevations in cell proliferation

**Fig 4:**
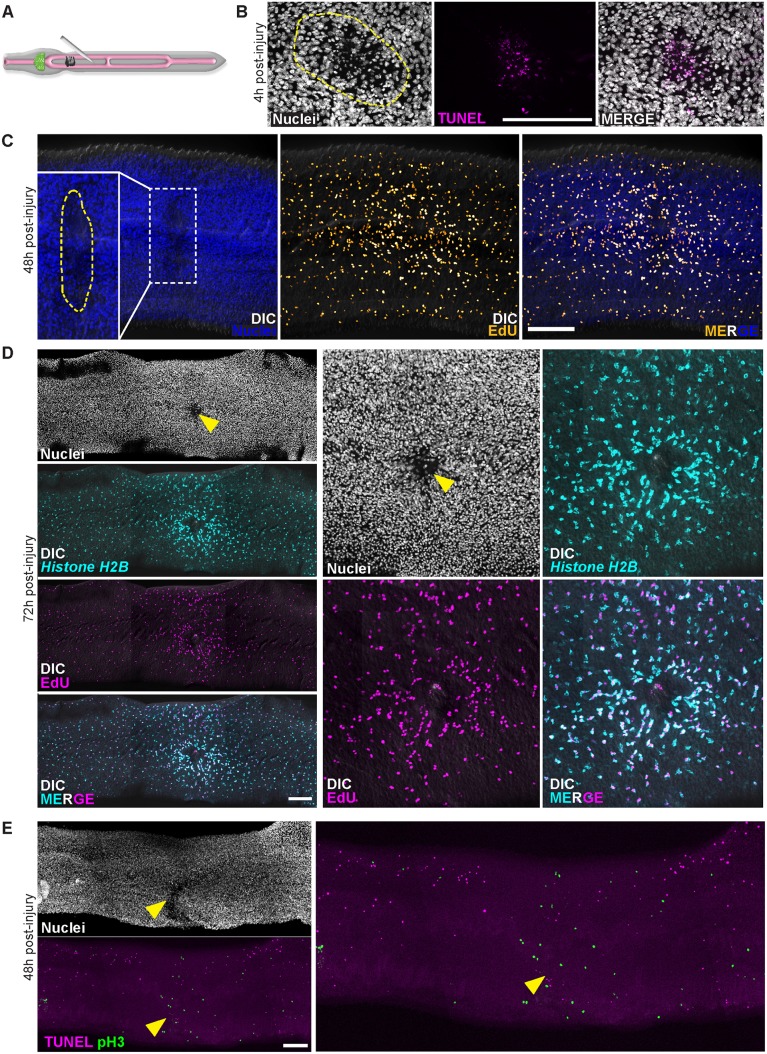
Physical injury initially induces cell death and increases in neoblast proliferation. (A) Cartoon showing strategy to injure worms. (B) 4 hours post-injury the number of TUNEL^+^ cells is increased at the site of injury. Dashed line approximates the site of injury. Images representative of n = 21/23 male parasites. (C) EdU-incorporating neoblasts accumulate near wound site at 48 hours post-injury (n = 18/19 parasites). Parasites were fixed after a 4 hour EdU pulse. Dashed line in inset approximates the site of injury. (D) *Histone H2B* ^+^ neoblasts at 72 hours post-injury are enriched around wound sites (n = 11/11 male parasites). Parasites were labeled with EdU for 4 hours at 48 hours post-injury and were fixed 24 hours later (72 hours post-injury). Arrowhead indicates approximate site of injury. (E) TUNEL staining for cell death and Phospho-Histone H3 (pH3) labeling for neoblasts in mitosis in animals at 48 hours post-injury. Mitotic neoblasts 48 hours post-injury are clustered at wound sites (n = 28/29 parasites), whereas the number of TUNEL^+^ cells are reduced in tissues adjacent to wound sites (n = 24/26 male parasites). Arrowhead indicates approximate site of injury. Scale Bars: 100 μm. D and E are titled images from multiple confocal stacks.

In diverse organisms (e.g., *Hydra* [22], *Drosophila* [23], planarians [19]) tissue injury induces apoptosis and precedes increases in stem cell proliferation [24]. Therefore, one attractive model to explain the simultaneous elevations of both neoblast proliferation and cell death observed in *cbp1(RNAi)* parasites could be that *cbp1* is required for the survival of various cell types in the worm (e.g., esophageal gland cells) and that death of these cells induces neoblast proliferation. Alternatively, *cbp1* could be acting in some cells (e.g., esophageal gland cells) to promote cell survival and acting independently in neoblasts to repress proliferation. To indirectly distinguish between these possibilities, and examine if tissue injury can induce both cell death and neoblast proliferation in schistosomes, we physically injured male parasites. For these experiments, parasites were immobilized on an agarose pad and poked with a sharpened tungsten needle (Fig 4A). Consistent with this injury regime inducing tissue damage and subsequent cell death in the worm, we noted substantial numbers of TUNEL^+^ nuclei at wound sites 4 hours post-injury (Fig 4B). We next examined injured parasites with neoblast and cell proliferation markers 48–72 hours following injury. Consistent with injury inducing neoblast proliferation, we noted accumulations of EdU-incorporating cells (Fig 4C) and *Histone H2B* ^+^ neoblasts (Fig 4D) surrounding wound sites at both 48 and 72 hours post-injury. Similarly, by immunofluorescence we noted increases in cells positive for M-phase specific marker Phospho-Histone H3 at sites adjacent to wounds (Fig 4E). Interestingly, examination of parasites at 48-hours post-injury by TUNEL staining found that although increases in cell death could often still be detected at the wound site, rates of cell death were depressed in the tissues immediately adjacent to wounds relative to the rest of the parasite (Fig 4E). This suggests that injury may repress physiological rates of cell death in tissues near wound sites. This repression of cell death may serve as a mechanism to preserve the function of tissues undergoing repair. Taken together, our data suggest that injury, and perhaps cell death, is capable of stimulating neoblast proliferation. Furthermore, these data suggest that schistosomes may be capable of utilizing neoblast-mediated tissue renewal to fuel tissue repair following injury.

### *cbp1* is essential for schistosome survival *in vivo*

**Fig 5:**
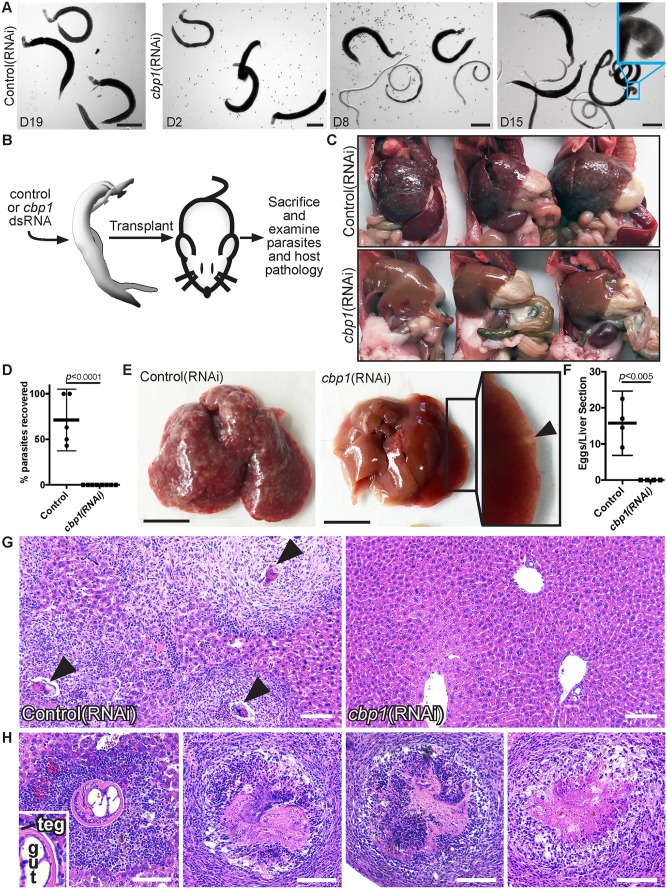
*cbp1* is essential for schistosome survival *in vivo*. (A) Images of control and *cbp1* RNAi-treated worms during *in vitro* culture. Until at least D19 of *in vitro* culture, control(RNAi) animals remain as male and female worm pairs, are firmly attached to the substrate, and maintain vigorous movement. By D8 of *in vitro* culture, *cbp1(RNAi)* animals become unpaired and fail to firmly attach to the substrate. Over time, movements of *cbp1(RNAi)* worms become less vigorous and oftentimes their heads curled ventrally (Cyan box, D15 time point). Cartoon depicting surgical procedure to examine the requirement for *cbp1* function *in vivo*. (C) Images of three mice that were transplanted with control or *cbp1* RNAi-treated parasites following hepatic vein perfusion. Unlike mice that received control(RNAi) worms, livers from mice receiving *cbp1(RNAi)* worms were normal-sized and the mice had few signs of infection. Plot showing quantification of the percent recovery of control and *cbp1* RNAi-treated parasites from mice. Each dot represents percent recovery from an individual mouse. Two separate sets of transplantations were performed with n = 5 mice for controls and n = 8 mice for *cbp1(RNAi)*. *p* < 0.0001, t-test. Representative livers from mice transplanted with control or *cbp1* RNAi-treated parasites. Livers from mice that received control(RNAi) parasites were large and contained large numbers of granulomas. Livers from mice receiving *cbp1(RNAi)* parasites were normal sized and contained very few granulomas. Few large granuloma-like structures were often found at the periphery of livers from mice that received *cbp1(RNAi)* worms (arrowhead in inset). Plot depicting number of schistosome eggs per liver section from mice transplanted with control or *cbp1* RNAi-treated parasites. Each dot represents the mean number of eggs counted from two liver sections from an individual mouse. n = 4 livers for both control and *cbp1* RNAi treatment groups. H&E staining of liver tissue from mice transplanted with control or *cbp1* RNAi-treated worms. Arrowheads point to eggs inside granulomas. Large granuloma-like masses in livers of mice from *cbp1*(RNAi) treatment group correspond to worms at various stages of degeneration. Left panel shows a male worm with a clearly identifiable tegument and intestine (labeled teg and gut in inset, respectively) surrounded by neutrophils and lymphocytes. As panels move to the right, worms appear to become structurally compromised and lesions contain more host fibroblasts, suggesting these lesions possess a more mature immune response to the parasites. Scale Bars: A,E 1 mm; G-H 100 μm.

Presumably due to elevations in cell death and declining tissue function, we observed that *cbp1(RNAi)* parasites became progressively sicker during *in vitro* culture (Fig 5A, S1 Movie). By D8, male and female *cbp1(RNAi*) parasites became unpaired and lost the ability to attach to the surface of the tissue culture dish (Fig 5A). By D15, parasite movement became uncoordinated and often times the heads of male worms curled ventrally (Fig 5A, S1 Movie). At D19, movement in *cbp1(RNAi)* parasites was limited to irregular and jerky motions (S1 Movie). The progressive decline in the vitality of parasites was not likely due to elevations in cell proliferation since irradiated *cbp1(RNAi)* parasites were indistinguishable from unirradiated *cbp1(RNAi)* parasites with regards to male-female pairing and attachment to the substrate (S1 Movie).

Given the complexity of the schistosome lifecycle and the lack of robust transgenic tools, few studies to date have examined adult schistosome gene function in the context of a mammalian host [25]. To explore if *cbp1* is essential for parasite survival *in vivo*, we coupled *in vitro* RNAi treatment with a procedure pioneered by Cioli in the 1970’s for the surgical transplantation of schistosomes into the mesenteric veins of rodent hosts [26]. For these experiments, 4 to 5 week old parasites were recovered from mice, treated for 4 days with control or *cbp1* dsRNA *in vitro*, and then surgically transplanted into the mesenteric veins of recipient mice (Fig 5B). At D26 post-transplantation, we euthanized the mice, performed hepatic portal vein perfusion, and measured both the percent recovery of transplanted parasites and extent of schistosome induced host pathology. In mice that received control(RNAi) worms, we noted hepatosplenomegaly consistent with the transplanted parasites establishing a productive infection (Fig 5C). Following hepatic portal vein perfusion, we recovered about 70% of the male control(RNAi) parasites originally transplanted (Fig 5D). In contrast to controls, mice receiving *cbp1(RNAi)* parasites did not display hepatosplenomegaly (Fig 5C) and we failed to recover any male parasites following hepatic portal vein perfusion (Fig 5D). We also noted obvious signs of egg-induced liver pathology in control(RNAi) recipient mice (Fig 5E); no evidence of egg-induced granuloma formation was observed in *cbp1(RNAi)* recipient mice (Fig 5E). Examination of histological sections from the livers of control and *cbp1(RNAi)* recipient mice confirmed that control parasites were capable of generating egg-induced pathology whereas no egg-induced inflammation was observed in *cbp1(RNAi)* recipient mice (Fig 5F and 5G).

Although we detected no signs of egg-induced inflammation, we did note large masses located at the periphery of the livers of *cbp1(RNAi)* recipient mice (Fig 5E, arrowhead). Examination of these livers in histological sections found these masses to be *cbp1(RNAi)* parasites trapped in the liver of these mice (Fig 5H). Observing these sections in more detail, we identified worms at several stages of deterioration: some parasites were relatively intact with an uninterrupted tegument (Fig 5H, left panel) whereas others were severely degenerated with virtually no organized schistosome tissues (Fig 5H, right panel). The composition of host cells surrounding the parasite, and the apparent maturity of the immunological response to the worms, correlated with the structural integrity of the worms. More intact worms were surrounded by large numbers of neutrophils and lymphocytes (indicative of early host response) whereas more degenerated worms were found in lesions encased in fibroblasts (indicative of a mature host response to the parasites). These data suggest *cbp1(RNAi)* parasites are incapable of establishing an infection. Based on what we observe *in vitro* (Fig 5A and S1 Movie), we hypothesize that within 4–5 days following transplantation these parasites lose the ability to attach to the host endothelium and are washed into the liver. In the liver, the health of the parasites continues to decline and they are susceptible to being killed by the host immune system, perhaps in a similar fashion as schistosomes treated with praziquantel *in vivo* [27]. Based on these data, we suggest that *cbp1* is essential for schistosome survival *in vivo*.

## Discussion

**Fig 6:**
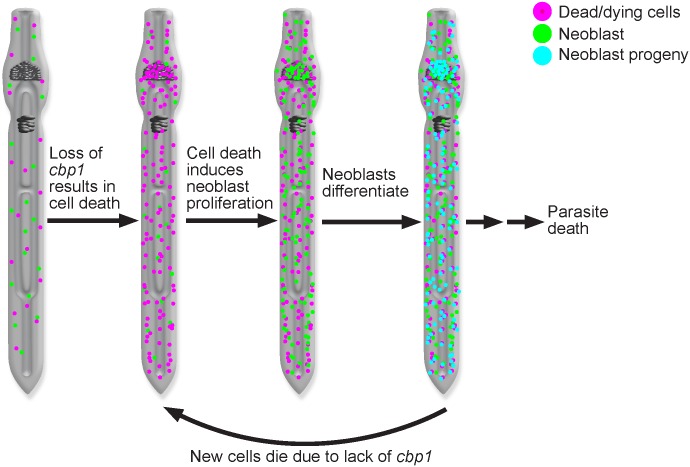
Model for observed consequences of loss of *cbp1*. Reduced *cbp1* function induces cell death and tissue degeneration. This triggers neoblast proliferation and an increased rate of neoblast differentiation to repair damaged tissues. Since *cbp1* levels remain reduced, tissues continue to degenerate, triggering more neoblast proliferation. Parasites eventually die due to defects in tissue function.

Aside from supporting new cell birth during the physiological turnover of tissues (e.g., the tegument [5]), we know relatively little about the roles that neoblasts play in the biology of adult schistosomes. Here, we report that reductions in *cbp1* levels result in simultaneous elevations of both cell proliferation and cell death. The esophageal glands were emblematic of this: apoptosis driven cell death was accompanied by massive accumulations of proliferative neoblasts. These observations suggested that neoblasts might be equipped to respond to lost or damaged tissues, an observation we confirmed by demonstrating that physical wounding induced proliferative neoblasts to accumulate around wound sites. Based on these data we suggest a model in which reduction of *cbp1* levels leads to cell death and tissue loss throughout the parasite (Fig 6). This cell loss is (directly or indirectly) sensed by neoblasts resulting in an increased rate of neoblast proliferation. Since we observe large increases in the number of cells expressing the neoblast progeny marker *tsp-2*, it is likely the neoblasts then differentiate to restore lost cells. Because *cbp1* levels remain depressed due to the effects of RNAi these newly differentiated cells die, inducing more neoblast proliferation. Tissue degeneration and the inability of neoblasts to restore tissue function eventually results in parasite death.

While physical injury induces schistosome neoblast proliferation, the precise role apoptosis and other types of cell death (e.g., necrosis) play in this process are not known. In the cnidarian *Hydra*, programmed cell death releases Wnt molecules that are required to induce stem cell proliferation and regeneration following amputation [22]. In *Drosophila*, genetic induction of apoptosis stimulates proliferation of intestinal stem cells [23]. In planarians, injury induces apoptosis although the requirement for cell death in fueling regeneration is not clear [19]. Therefore, dying cells in schistosomes may directly signal to induce neoblast proliferation. Alternatively, a myriad of other factors (e.g., loss of tissue integrity and/or loss of cell-cell contacts) may stimulate neoblast proliferation. As tools to study schistosome cell death mature, it should be possible in the future to determine precisely how apoptosis influences neoblast behavior.

Mammalian *cbp1* homologs serve as transcriptional co-activators linking transcription factors to the core transcriptional machinery [11]. These mammalian *cbp1* relatives also possess acetyltransferase activity and can acetylate a variety of substrates including histones and non-histone proteins [11]. Whether these activities of *cbp1* are important for maintaining schistosome cellular viability is not presently clear. However, previous studies have shown that pharmacological inhibition of histone deacetylase activity induces apoptosis in larval schistosomes [28, 29]. Thus, not unexpectedly, maintaining normal chromatin structure is likely important for schistosome cellular survival. Since *cbp1* possesses histone acetyltransferase activity *in vitro* [10], the cell death induced by *cbp1* may be due to alterations in chromatin landscape in certain cell types. Further exploration of chromatin-modifying enzymes may represent fertile ground for the development of novel therapeutics.

Here, we combine a previously described method for the surgical transplantation of schistosomes and RNA interference to demonstrate that *cbp1* is required for parasite survival *in vivo*. Not only do these studies validate the potential to target *cpb1* therapeutically, they provide a novel methodology to explore the functions of schistosome genes *in vivo*. A potentially useful application of this approach is for studies of schistosome reproduction. Since schistosome reproduction ceases within one week of *in vitro* culture [20], this approach could help identify genes required for the development and maintenance of the schistosome reproductive system. One potential limitation of this approach is the persistence of the effects of RNAi. Although the effects of RNAi have been reported to last for several weeks in larval schistosomes *in vitro* [30], how this translates to older parasites *in vivo* is not known. However, as tools to manipulate the schistosome genome (i.e., transgenic expression and genome editing) continue to mature, we suggest surgical transplantation could become an invaluable tool to explore gene function *in vivo*.

Our observation that injury is met with increases in neoblast proliferation indicates schistosomes may possess the capacity to regenerate following certain types of injury *in vivo*. The regenerative potential of schistosomes has not been extensively characterized and conflicting reports exist. Senft and Weller reported that schistosomes amputated during recovery from mice were capable of regenerating new tails *in vitro* [31]. However, this conflicts with another account where *in vitro* cultured worms were capable of rapidly healing wounds, but incapable of regeneration [32]. Thus, the ability of schistosomes to perform whole-body regeneration (i.e., regenerating new heads and/or tails) is unresolved and may be a function of culture conditions and the nature of the injury. What is less controversial is the ability of schistosomes to repair tissues following *in vivo* exposure to sublethal doses of the anthelminthic drug praziquantel [33]. Thus, future studies exploring roles for neoblasts in tissue repair, following a variety of injuries (e.g., amputation and drug treatment) and in a variety of culture conditions, are necessary and could have important implications for understanding the longevity and resilience of these parasites *in vivo*.

## Materials and methods

### Ethics Statement

In adherence to the Animal Welfare Act and the Public Health Service Policy on Humane Care and Use of Laboratory Animals, all experiments with and care of vertebrate animals were performed in accordance with protocols approved by the Institutional Animal Care and Use Committee (IACUC) of the UT Southwestern Medical Center (protocol approval number APN 2014–0072).

### Parasite Acquisition and Culture

Adult *S*. *mansoni* (6–8 weeks post-infection) were obtained from infected female mice by hepatic portal vein perfusion with 37°C DMEM (Sigma-Aldrich, St. Louis, MO) plus 10% Serum (either Fetal Calf Serum or Horse Serum) and heparin. Parasites were cultured as previously described [5]. Unless otherwise noted, all experiments were performed with male parasites.

### Molecular Biology

cDNAs used for *in situ* hybridization and RNA interference were cloned as previously described [34]. Quantitative PCR analyses were performed as previous described [5]. Oligonucleotide sequences are listed in S1 Table.

### RNA interference, parasite labeling, and imaging

EdU labeling, whole-mount in situ hybridization and fluorescence in situ hybridization analyses were performed as previously described [4, 5]. For RNAi experiments, 5–10 freshly perfused male parasites (either as single worms or paired with females) were treated with 30 μg/ml dsRNA for 4 days in Basch Media 169 [35]. dsRNA was generated by *in vitro* transcription [4] and was replaced every day. As a negative control for RNAi, we used a non-specific dsRNA containing two bacterial genes [4]. Sequences used for dsRNA synthesis are listed in S2 Fig. For irradiation of RNAi-treated parasites, worms were exposed to 100 Gy of Gamma Irradiation using a J.L. Shepard Mark I-30 Cs^137^ source. Lectin labeling was performed as previously described [21]. For TUNEL labeling, parasites were fixed for 4 hours in 4% Formaldehyde in PBS + 0.3% Triton X100 (PBSTx), dehydrated in methanol, and stored at -20°C. Parasites were subsequently rehydrated with PBSTx, permeabilized with 20ug/ml Proteinase K (Invitrogen, Carlsbad, CA) in PBSTx for 45 min, and post-fixed with 4% Formaldehyde in PBSTx. Following fixation parasites were processed for TUNEL labeling using the In situ BrdU-Red DNA Fragmentation (TUNEL) Assay Kit (Abcam). For this procedure, post-fixed worms were briefly incubated in the kit provided “wash” buffer, incubated in “DNA labeling solution” (2 to 3 male worms per 50 ul) for 4 hours at 37°C, rinsed twice in PBSTx, blocked with “FISH Block” (0.1 M Tris pH 7.5, 0.15 M NaCl and 0.1% Tween-20 with 5% Horse Serum and 0.5% Roche Western Blocking Reagent [36]), and incubated overnight in Anti-BrdU-Red Antibody (1:20) in “rinse buffer”. After several PBSTx washes, worms were either mounted on slides in Vectashield (Vector Labs, Burlingame, Ca) or further processed for immunofluorescence or lectin labeling. For immunofluorescence, permeabilized worms were blocked in FISH Block and incubated overnight at 4°C in Anti-Phospho-Histone H3 (Ser10) (Rabbit mAB, D2C8, Cell Signaling) diluted 1:1000 in FISH block. Following 6 x 1 hour washes in PBSTx worms were incubated overnight at 4°C in Goat anti-Mouse IgG secondary antibody conjugated to AlexaFluor 488 diluted in FISH block (Thermo Fisher). Following several washes in PBSTx, parasites were mounted on slides in Vectashield.

Confocal imaging of fluorescently labeled samples and brightfield imaging (i.e, whole-mount in situ hybridizations and histological sections) were performed using a Zeiss LSM700 Laser Scanning Confocal Microscope or a Zeiss AxioZoom V16 equipped with a transmitted light base and a Zeiss AxioCam 105 Color camera, respectively. All images of fluorescently-labeled samples represent maximum intensity projections. To perform counts of EdU^+^ and TUNEL^+^ cells, cells were manually counted in maximum intensity projections derived from confocal stacks; to normalize between samples cell counts were divided by the total volume of the stack in μm^3^. All plots and statistical analyses were performed using GraphPad Prism.

### Worm Injury

For injury, worms were gently pipetted onto the surface of a 35 mm Petri dish filled with solidified 4% agarose diluted in H_2_O. After removal of excess liquid, worms were perforated with a sharpened tungsten needle. The impaled parasites were then carefully removed from the needle into fresh media using a pipette tip. As a control, “mock” injured parasites were similarly transferred to Petri dishes but were not injured; we observed no changes in cell death or cell proliferation in these parasites.

### Surgical Transplantation of schistosomes

Methods for surgical transplantation of schistosomes are based on a procedure originally developed for hamsters [26]. 4 to 5 days prior to surgery parasites 4–5 weeks post-infection were recovered from mice and treated with 30 μg/ml dsRNA for 4 days in Basch Media 169 [35] as previously described [4]. Media and dsRNA were changed daily. Before mice were anesthetized, 8 male parasites (either paired or unpaired with female, see below) were sucked into a 1ml syringe, the syringe was fitted with a custom 25G extra thin wall hypodermic needle (Cadence, Cranston, RI), the air and all but ~300 μL of media were purged from the needle, and the syringe was placed needle down in a test tube to settle the parasites to the bottom of the syringe. We attempted to inject male/female worm pairs, but it was not always clear if females were present in the gynecophoral canal. Therefore, each injection also included a few unpaired female parasites to ensure maximal potential for mating. Once the syringe was loaded with parasites, young male Swiss Webster mice (~25–30G) were anesthetized with Isoflurane using a vaporizer system equipped with both an induction chamber and nose cone. Abdomens of anesthetized mice were shaved and the area was sterilized with three alternating scrubs with betadine and ethanol. A single longitudinal incision (~1.5 cm) centered on the navel was made to expose the intestines. A sterile piece of gauze with a 2 cm slit in the center was dampened with sterile saline and placed over the incision. The intestines were gently fed through the gauze to expose the large vein running along the cecum. The intestines were kept damp throughout the entire procedure with sterile saline. Making sure the bevel of the needle remained facing down, the worms were injected into the cecal vein. To avoid hemorrhage, prior to removing the needle a small piece of hemostatic gauze (Blood Stop) was placed over the injection site. As the needle was removed, gentle pressure was applied to the injection site. Once bleeding stopped (~1–2 minutes) the hemostatic gauze was removed and the intestines returned into the abdominal cavity. The cavity was filled with sterile saline and abdominal muscles and skin were sutured (Maxon, Absorbable Sutures, Taper Point, Size 4–0, Needle V-20, ½ Circle). Following wound closure, mice received a single subcutaneous dose of buprenorphine for pain (30 μl of 1 mg/ml) and were allowed to recover on a warm heating pad. After transplant, needles were flushed with media to determine how many parasites had been injected into each mouse. Mice were group housed and individual mice were tracked by marking their tails with a permanent marker. On day 26 post-transplantation mice were sacrificed and perfused to recover parasites. Male and female parasites were counted and livers were removed and fixed for 30–40 hours in 4% formaldehyde in PBS. The percentage parasite recovery was determined by dividing the number of male worms transplanted by the number of male parasites recovered following perfusion. Counting male parasites was the most informative since the initial number of female parasites was not accurately quantified (see above). Livers from individual mice were sectioned and processed for Haematoxylin and Eosin staining by the UT Southwestern Molecular Pathology Core.

## Supporting Information

S1 Fig *cbp1(RNAi)* treatment specifically reduces *cbp1* transcript levels. (A) Cartoon of *cbp1* cDNA (top) and cDNA regions (in bp) targeted by two independent RNAi constructs (pJNC9.1 and pJC259.1). pJNC9.1 contains a cDNA fragment that spans from 2057bp to 3060 bp of the *cbp1* cDNA. pJC259.1 contains a cDNA fragment that spans from 4838bp to 5015bp and 5621bp to 5839bp of the *cbp1* cDNA; this cDNA appears to be alternatively spliced relative to the *cbp1* gene model. Full-length sequences of these cDNA fragments are found in S2 Fig.
(B) Expression of *cbp1* in control and *cbp1(RNAi)* parasites relative to a proteasome subunit (Smp_056500) as measured by qPCR. *cbp1(RNAi)* treatment using dsRNA produced from pJNC9.1 results in a statistically significant reduction in *cbp1* mRNA levels (p < 0.025, t-test, n = 3 biological replicates from male parasites with their heads and testes removed). Similar levels of knockdown were observed with pJC259.1. Error bars represent 95% confidence intervals.
(C) EdU labeling in control and *cbp1(RNAi)* parasites treated with dsRNA generated from pJC259.1 at D13 of RNAi. *cbp1(RNAi)* using dsRNA produced from pJC259.1 resulted in elevations in cell proliferation similar to RNAi treatment using dsRNA from pJNC9.1. Parasites were pulsed with EdU overnight prior to fixation. Arrowhead indicates approximate position of esophageal gland where we often noted large numbers of proliferative neoblasts.
(TIF)

S2 Fig Nucleotide sequences used for RNAi. (A-C) Shown are sequences used as templates to generate dsRNA for (A) control(RNAi) (B) *cbp1(RNAi)* pJNC9.1, and (C) *cbp1(RNAi)* pJC259.1.
(TIF)

S1 Table Oligonucleotide sequences used in this study. (XLSX)

S1 Movie Movies showing behavior of control and *cbp1(RNAi)* parasites at various time points following the initial dsRNA treatment *in vitro*. The health of *cbp1(RNAi)* parasites declines beginning around D8 of RNAi when parasites become unpaired and fail to attach to the surface of the culture dish. This deterioration of the health of *cbp1(RNAi)* parasites is not influenced by stem cell number since irradiated *cbp1(RNAi)* parasites experience similar declines as unirradiated worms.
(MP4)
