# Supplementary figures and images for: Tissue Degeneration following Loss of Schistosoma mansoni cbp1 Is Associated with Increased Stem Cell Proliferation and Parasite Death In Vivo
Source: PLoS Pathog. 2016 Nov 3;12(11):e1005963. doi: 10.1371/journal.ppat.1005963 (PMC5094730; doi:10.1371/journal.ppat.1005963)

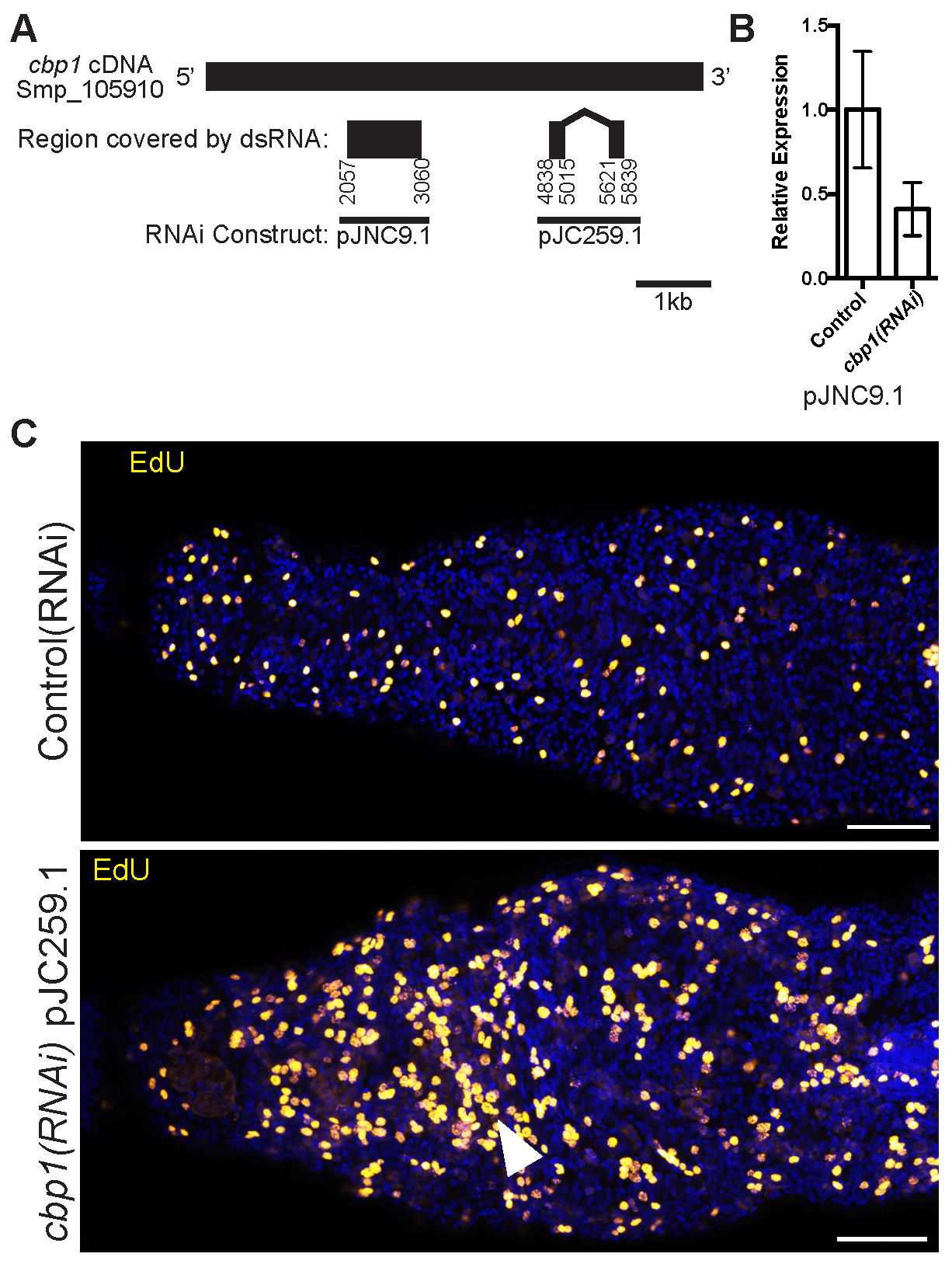

Supplement: S1 Fig — (A) Cartoon of cbp1 cDNA (top) and cDNA regions (in bp) targeted by two independent RNAi constructs (pJNC9.1 and pJC259.1). pJNC9.1 contains a cDNA fragment that spans from 2057bp to 3060 bp of the cbp1 cDNA. pJC259.1 contains a cDNA fragment that spans from 4838bp to 5015bp and 5621bp to 5839bp of the cbp1 cDNA; this cDNA appears to be alternatively spliced relative to the cbp1 gene model. Full-length sequences of these cDNA fragments are found in S2 Fig. (B) Expression of cbp1 in control and cbp1(RNAi) parasites relative to a proteasome subunit (Smp_056500) as measured by qPCR. cbp1(RNAi) treatment using dsRNA produced from pJNC9.1 results in a statistically significant reduction in cbp1 mRNA levels (p < 0.025, t-test, n = 3 biological replicates from male parasites with their heads and testes removed). Similar levels of knockdown were observed with pJC259.1. Error bars represent 95% confidence intervals. (C) EdU labeling in control and cbp1(RNAi) parasites treated with dsRNA generated from pJC259.1 at D13 of RNAi. cbp1(RNAi) using dsRNA produced from pJC259.1 resulted in elevations in cell proliferation similar to RNAi treatment using dsRNA from pJNC9.1. Parasites were pulsed with EdU overnight prior to fixation. Arrowhead indicates approximate position of esophageal gland where we often noted large numbers of proliferative neoblasts. (TIF) [file ppat.1005963.s001.tif]

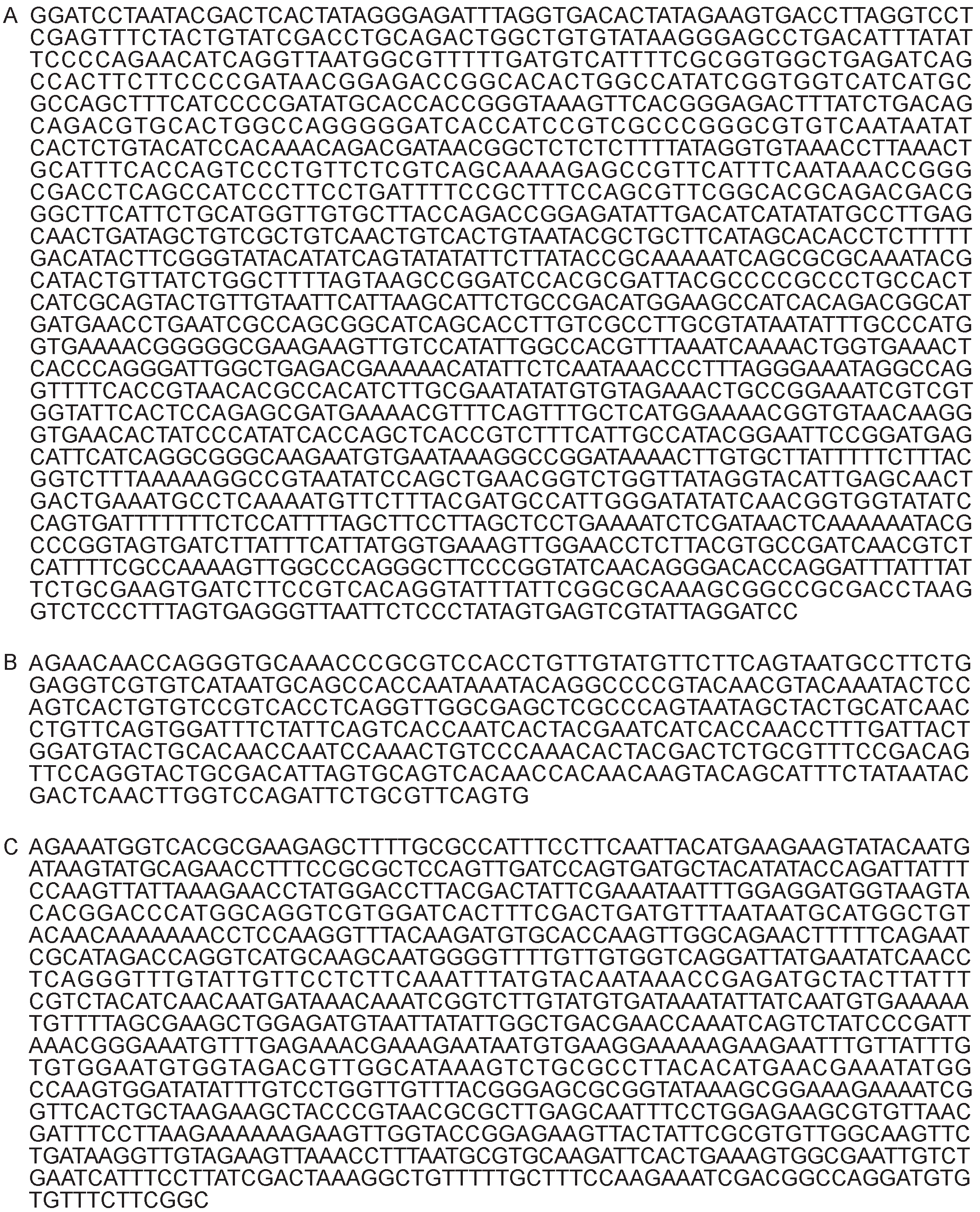

Supplement: S2 Fig — (A-C) Shown are sequences used as templates to generate dsRNA for (A) control(RNAi) (B) cbp1(RNAi) pJNC9.1, and (C) cbp1(RNAi) pJC259.1. (TIF) [file ppat.1005963.s002.tif]
